# Beyond Blood and Urine: Decoding the Human Chemical Exposome across Matrices and Time

**DOI:** 10.1021/acs.est.6c06509

**Published:** 2026-09-18

**Authors:** Adrià Sunyer-Caldú

**Affiliations:** † Department of Environmental Science, Science for Life Laboratory, Stockholm University, Stockholm 10691, Sweden; ‡ National and Kapodistrian University of Athens, Panepistimioupolis, Zographou, Athens 15771, Greece

**Keywords:** chemical exposomics, high-resolution mass spectrometry (HRMS), longitudinal monitoring, multi-matrix integration, precision environmental health

## Abstract

Environmental chemicals contribute substantially to the global burden of chronic disease, yet for most compounds now detectable in human biological samples, causal links to specific health outcomes remain poorly established. HRMS can profile thousands of chemical features simultaneously in a single sample. What has not kept pace is study design. No investigation has yet combined external environmental source monitoring, multi-matrix internal biomonitoring, and toxicokinetic normalization within a single longitudinal framework. This Perspective argues that closing this design gap is the prerequisite for strengthening causal evidence in environmental health research and proposes an operational framework resting on three contributions: a multi-matrix sampling imperative extending the biological inventory beyond blood and urine to accumulation archives, developmental matrices, and external environmental media; a Unified Exposure Scale translating measurements from heterogeneous matrices onto a common toxicokinetically normalized dosimetric axis; and a two-track hazard prioritization pipeline for the largely uncharacterized molecular dark matter of the exposome. As the Global Human Exposome Project defines its operational infrastructure, these design principles offer a concrete path toward identifying chemical disease drivers before clinical manifestations.

## Graphical abstract

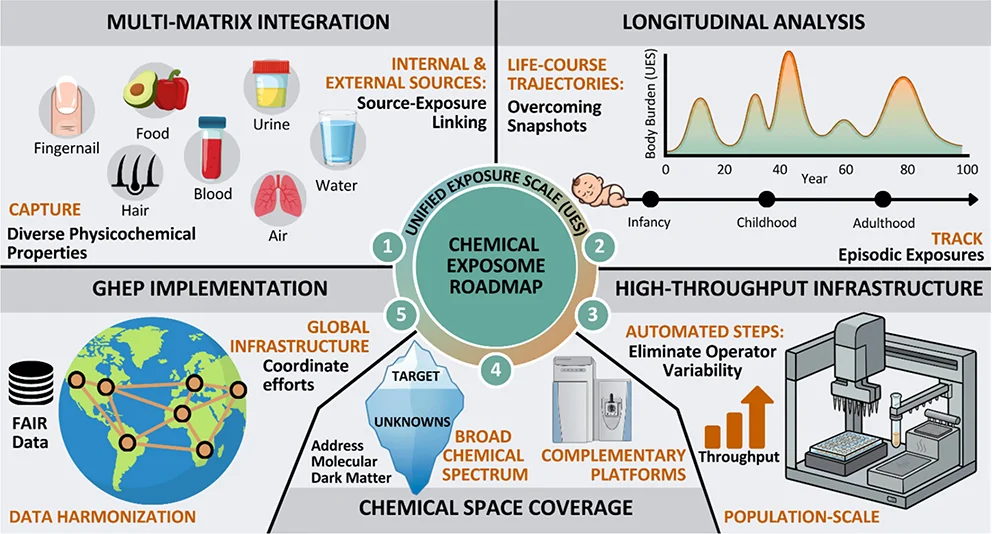

## Introduction

Environmental chemicals contribute to a substantial fraction of the global burden of chronic disease,^1^ yet for the vast majority of compounds now detectable in human biological samples, causal links to specific health outcomes remain poorly established. The exposome was proposed two decades ago to provide the framework needed to address this,^2^ and the field has since matured into a recognized driver of precision health research.^3^ What has not kept pace is study design. HRMS and nontarget screening (NTS) can simultaneously profile thousands of unknown chemical signals—commonly referred to as features—in a single biological sample,^4^ but most exposure assessments still compress a lifetime of chemical encounters into one blood draw taken at one point in time.^5^ The problem with that approach is not only sensitivity—it is that exposure never stops changing.^6^ A pesticide spike in March, cleared from blood within days, leaves no trace in a June sample. No study built around that June sample will ever know it happened (Figure 1). Environmental chemicals account for a large fraction of the chronic disease burden, yet the proportion attributable to precisely these transient, episodic, or mixture exposures—the ones cross-sectional designs structurally cannot capture—has never been quantified. That is the design gap this perspective addresses.^7^

**Figure 1:**
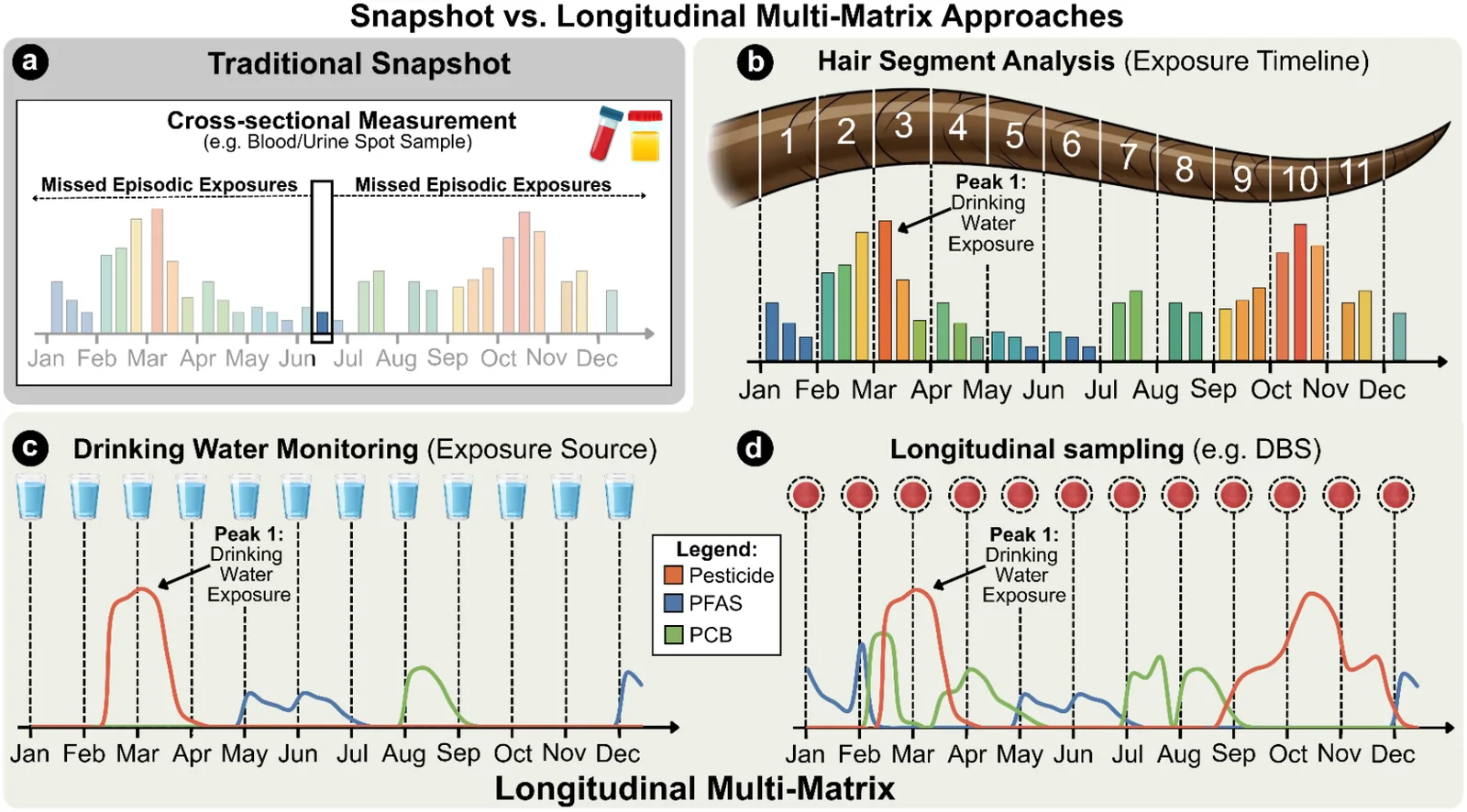
Snapshot versus longitudinal multi-matrix approaches in chemical exposomics. (a) Point-in-time sampling illustrates the snapshot paradigm: a June blood draw misses a pesticide spike that occurred in March and cleared within days. (b) Segmental hair analysis resolves that March event retrospectively, providing a chronological archive of accumulated exposure at monthly resolution. (c) Environmental source monitoring—here, drinking water—documents the external chemical environment and enables source attribution. (d) High-frequency microsampling tracks internal dose trajectories continuously across complex chemical mixtures including pesticides, PFAS, and PCBs. Combining environmental monitoring (c) with retrospective biological archives (b) and prospective longitudinal sampling (d) allows reconstruction of the source-to-dose continuum.

Inside the body, blood and urine have dominated biomonitoring because standardized collection procedures exist and they provide accessible systemic data on internal dose.^8^ However, each matrix only reflects the chemicals it can retain under its specific physiological conditions.^9^ Hair records months of accumulated exposure for certain compound classes;^10^ adipose tissue holds lipophilic compounds that blood has already cleared;^11^ the chemical record transferred from mother to child is only accessible in placenta and cord blood;^12^ feces connects the chemical environment to the gut microbiome. Standardized NTS protocols for most of these matrices do not yet exist,^13^ but the appropriate response is to develop them, not to continue measuring only what is already convenient.

Outside the body, air,^14^ water,^15^ food,^16^ dust,^17^ and community-level proxies such as sewage sludge^18^ document where exposures originate.^19^ Wearable passive samplers and portable active sensors extend this further,^20,21^ resolving personal-level chemical burden in the specific environments where people actually spend their time. In practice, however, this external monitoring is almost never combined with internal biospecimen data.^22^ Without that integration, internal biomarker concentrations cannot be linked back to their sources, and source attribution is precisely what regulators and clinicians need to act.^23^

Gao et al. combined longitudinal personal external monitoring with internal chemical exposome and multi-omics profiling in a single participant.^24^ Jiang et al. maintained external chemical surveillance across hundreds of days in a small cohort.^25^ Sdougkou et al. characterized the temporal dynamics of the blood chemical exposome longitudinally in a wellness cohort.^6^ None combined diverse external source matrices, multi-matrix internal biomonitoring, and toxicokinetic normalization simultaneously within a single longitudinal design. Each component is dependent on the others to produce interpretable results. External monitoring identifies what is present in the environment but does not provide an internal dose. Internal biomonitoring quantifies body burden but cannot identify the source: urinary PAH metabolites from traffic emissions and from indoor cooking are analytically indistinguishable, yet the regulatory responses that they require are completely different. Toxicokinetic normalization converts raw matrix concentrations into a common dosimetric unit, but without concurrent external data the resulting dose estimate has no source attached. Studies resolving one dimension at a time produce partial answers.

Recent perspectives and viewpoints have mapped the field’s remaining gaps with unusual clarity. Martin^26^ argues that data quality and transparency are the priority, and design commitments made now will shape what the field can deliver for decades. Miller et al.^27^ call for integrating chemical measurement with process-based models of internal dose. Gago-Ferrero et al.^28^ identify longitudinal multi-matrix profiling combined with environmental surveillance as the most pressing methodological frontier. Belova et al.^29^ point to single-matrix, single-timepoint designs as the principal bottleneck on mechanistic interpretation. Vermeulen and Safarlou^30^ argue that proposals for a Human Exposome Project remain operationally insufficient without coordinated measurement infrastructure. Schymanski et al.^31^ call for open, collaborative, discovery-driven exposomics built on FAIR data sharing and standardized annotation workflows as the foundation for cross-study comparability. What remains less defined is the study design that makes those ambitions operational—which matrices to sample, how to normalize measurements across them, and how to connect external sources to internal dose within a single longitudinal framework. This perspective proposes that framework (Figure 2) and argues it represents the minimum design condition for strengthening the source-to-dose relationships that causal inference in exposome research requires, and for advancing the goals of the Global Human Exposome Project (GHEP).^32^

**Figure 2:**
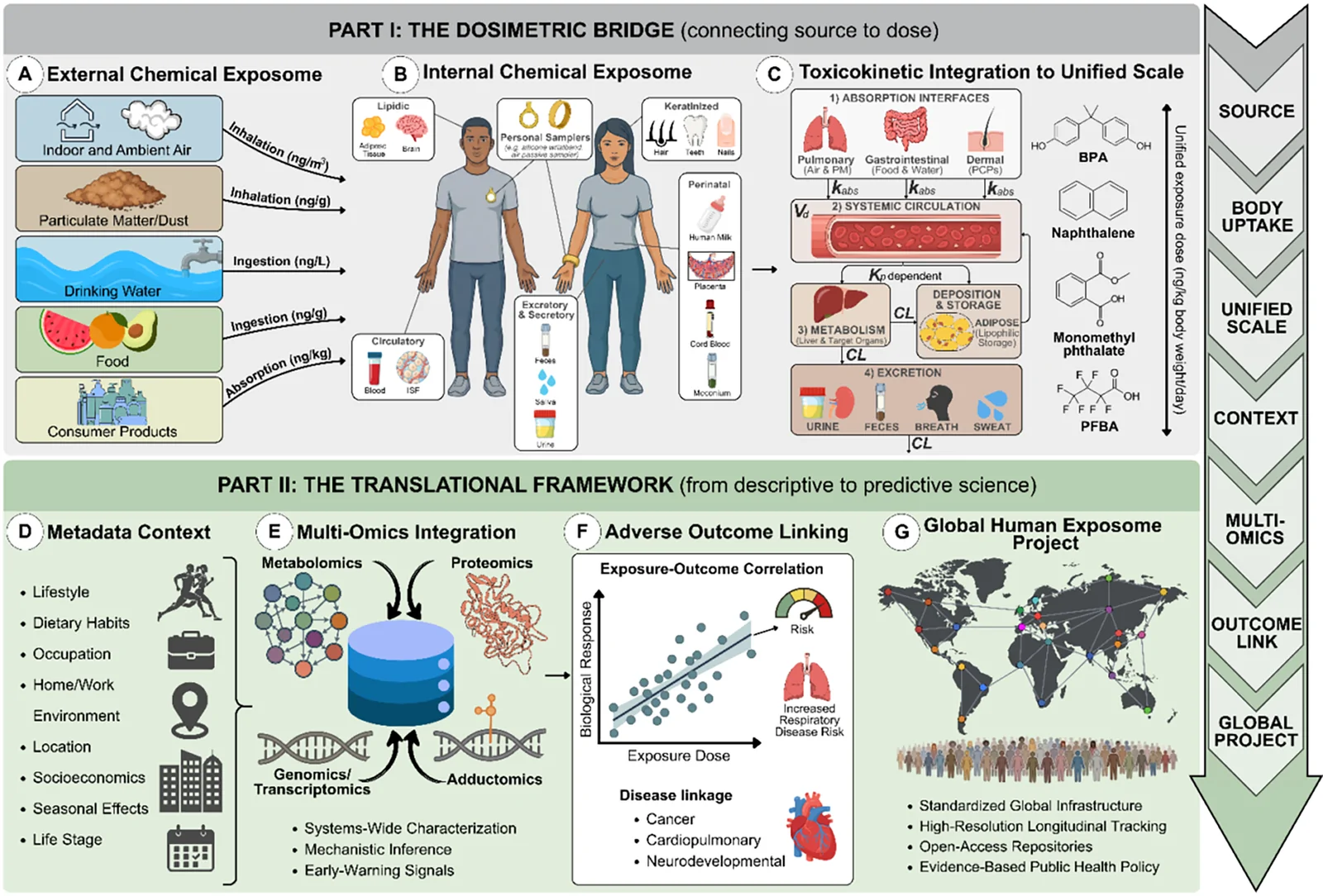
Proposed framework for comprehensive characterization of the human chemical exposome. (A) External chemical exposome: primary exposure media including air, dust, water, food, and consumer products, with associated entry routes. (B) Internal chemical exposome: biomonitoring across a diverse biological inventory spanning long-term accumulation matrices (teeth, hair, nails), excretory matrices (feces, sweat, breath), and maternal-fetal matrices (placenta, cord blood, meconium). (C) Toxicokinetic integration: PBPK-driven translation of raw matrix concentrations into a unified internal dose (ng/kg/day) using absorption (*k*_abs_), distribution (*V*_d_), partitioning (*K*_p_), and clearance (CL) parameters. (D) Metadata context: individual lifestyle, environment, and life-stage data. (E) Multi-omics integration: synthesis of exogenous chemical features with endogenous molecular profiles including metabolomics, proteomics, genomics, and adductomics. (F) Adverse outcome linking: correlation of internal dose with biological perturbation and clinical risk. (G) Global Human Exposome Project: international infrastructure, data harmonization, and policy translation. The vertical arrow illustrates the source-to-intervention workflow.

## The Multi-Matrix Imperative: Connecting the Environment to the Individual

Blood and urine became the standard matrices of human biomonitoring for good reasons—they are accessible, their collection is standardized, and their toxicokinetics are reasonably well understood.^8^ Both remain central to any biomonitoring design. What they cannot do is cover the full chemical inventory. Each matrix retains only the chemicals whose physicochemical properties allow them to accumulate there under specific physiological conditions.^9^ A cross-sectional sample taken days after a phthalate spike will miss everyone whose exposure cleared before the clinic visit, since short-lived pollutants are excreted within hours.^33^ If the effect is large enough, an association may still emerge—but the detectable exposure range is narrower than the true one, and effect sizes will be underestimated accordingly. Lipophilic chemicals partition into adipose tissue and are largely absent from peripheral blood,^11^ so blood measurements produce a systematic underestimate of total body burden for persistent organics. Volatile and particle-bound compounds have poor aqueous solubility and are barely represented in liquid matrices at all.

The solution is to sample from more than one biological compartment. Saliva, sweat, and feces extend coverage to excretory matrices, with feces providing a window into the microbiome-chemical interface that blood cannot access.^34^ Placenta, cord blood, and human milk capture chemical transfer during developmental windows of particular biological sensitivity.^12^ Segmental hair,^200^ nails, and deciduous teeth function differently— not as snapshot matrices but as chronological archives, preserving monthly-resolution exposure records across years of accumulation.^10^ Cerebrospinal fluid, where accessible, gives direct access to central nervous system chemical burden that peripheral sampling misses entirely,^35^ and microneedle-based interstitial fluid sampling allows continuous monitoring of the chemical environment at the cellular level.^36^ None of these matrices replaces blood or urine; each resolves chemical signals the others cannot^29^ (Figure 3). Belova et al.^29^ demonstrate this directly, showing that single-matrix, single-timepoint designs remain the principal bottleneck on mechanistic interpretation even when analytical methods are state of the art.

**Figure 3:**
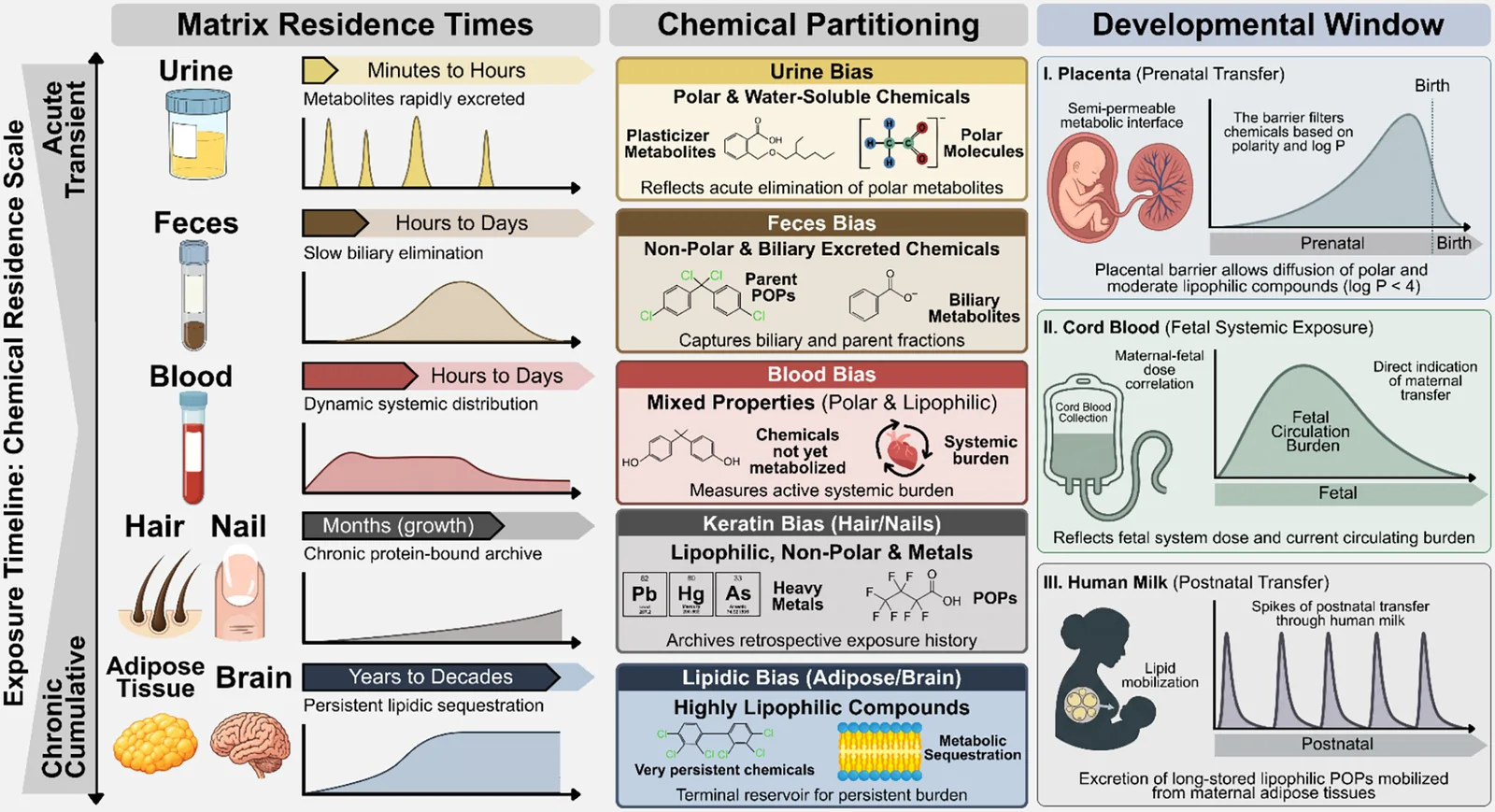
Temporal and physicochemical biases in the internal exposome. Chemical partitioning between aqueous, proteinaceous, and lipidic matrices dictates residence times (left), drives selective sequestration across biological compartments (middle), and determines the window of intergenerational chemical transmission (right). The figure distinguishes acute transient signals from chronic cumulative archives and illustrates maternal-fetal transfer kinetics across prenatal, fetal, and postnatal developmental windows.

Expanding the matrix inventory is only useful if the analytical methods applied to each matrix are sensitive enough to detect what is biologically present. Detection limits vary substantially across matrices, platforms, and laboratories,^4,13,37,38^ and complex backgrounds in feces, hair, and adipose tissue raise effective detection thresholds relative to plasma or urine. Targeted methods partially address this through selective extraction and acquisition strategies, achieving orders-of-magnitude sensitivity gains over untargeted workflows—but at the cost of chemical breadth. For most human matrices beyond blood and urine, sufficiently sensitive, robust, and reproducible untargeted methods have not yet been established. Instrument advances are narrowing this gap: ion mobility separation,^39^ hybrid acquisition modes, and faster scan speeds across platforms including the Orbitrap Astral, timsTOF Apex, Excedion, MRT P10, and ZenoTOF 8600 are progressively improving untargeted sensitivity and throughput. Analytical validation across the broader matrix inventory remains an open task.

Additionally, not all of these matrices are equally accessible. Blood, urine, hair, nails, saliva, and feces can be collected repeatedly from free-living participants, and these form the practical backbone of any longitudinal design. CSF, adipose tissue, teeth, placenta, and cord blood are clinically or opportunistically collected—valuable for specific research questions and retrospective reconstruction, but not realistic targets for routine longitudinal sampling in population cohorts. The longitudinal vision described here depends on the first tier. The second tier extends it where the opportunity exists or the research question demands it.

Equally important is the external chemical environment. Most biomonitoring studies measure internal concentrations without incorporating source data from air, water, dust, or food,^19,22^ which means internal biomarker levels cannot be traced back to their origins. Urinary PAH metabolites indicate exposure, but traffic emissions, indoor combustion, and contaminated food all produce similar profiles.^17^ The source remains speculative. The consequence is not just incomplete science—it is science that cannot support intervention. Without knowing the source, a regulator has no lever to pull. Integrating external monitoring with internal biospecimen data is not an analytical refinement^40^ it is what makes the data actionable in the first place. Gago-Ferrero et al.^28^ identify this integration—combining environmental surveillance with longitudinal multi-matrix biomonitoring—as the field’s most pressing methodological frontier.

When resources are limited, the priority combination is blood and urine for systemic burden and acute excretion, plus hair for retrospective baseline.^200^ These three together cover acute transient signals, chronic cumulative burden for certain compound classes, and the pre-study exposure history—at relatively low cost per participant using microsampling. External monitoring and additional matrices can be layered in as budget allows, but this core combination already addresses the most common sources of exposure misclassification in current cross-sectional designs. Additionally, direct environmental sampling carries logistical and cost demands^13^ that indirect approaches can complement. Residential questionnaires,^41^ occupational histories,^41^ and Geographic Information System (GIS)-based spatial models^19^ which link individual addresses to environmental data layers such as traffic density, green space, or industrial emissions—provide scalable exposure proxies with demonstrated correlations to internal biomarker levels, and are particularly useful at population scale. Where direct sampling is feasible, however, it remains the more informative option for source attribution—and the heterogeneity challenges associated with matrices such as dust are addressable through standardized collection and extraction protocols^42^ rather than reasons to avoid them.

Combining measurements across these matrices creates a comparability problem that has to be addressed explicitly. Concentrations in air (μg/m^3^), blood (ng/mL), urine (ng/L), and hair (ng/g) reflect different exposure windows and degrees of biological integration, and cannot be directly pooled or compared (Figure 3). This perspective proposes a Unified Exposure Scale (UES)—a toxicokinetically normalized metric in ng/kg body weight/day—that places measurements from all matrices on a common dosimetric axis using fugacity-based normalization—an approach that estimates chemical partitioning between biological compartments based on thermodynamic equilibrium principles—or high-throughput physiologically based pharmacokinetic (PBPK) models.^43–45^ Where matrices integrate exposure over different time scales, a dynamic PBPK framework^43^ works backward through each matrix’s kinetics to reconstruct the original exposure pattern, recovering pulses that would otherwise be lost when comparing measurements taken on different biological time scales.^27^ For compounds with characterized toxicokinetic parameters, the translation is robust; for the majority of NTS-detected features where those parameters are absent, property prediction from molecular structure allows approximate normalization^46^ sufficient to rank exposure magnitudes and prioritize features for follow-up without requiring reference standards.

Consider a cohort exposed to PFOA through contaminated drinking water and household dust. Blood concentrations alone suggest similar internal burden across participants. When external monitoring data are fed into a PBPK model alongside hair and urine measurements, the UES reveals that participants with near-identical blood levels have meaningfully different source contributions^47,48^ in some, drinking water dominates estimated daily intake; in others, dust ingestion and dermal contact prevail. The difference matters: water contamination calls for infrastructure intervention, dust exposure for indoor source identification. A single blood measurement cannot make that distinction. The UES does not eliminate uncertainty—PBPK parameters for many compounds remain approximate—but it shifts the analysis from describing internal burden to estimating where it comes from.

Because different matrices record exposure over different time scales—blood reflects days, hair reflects months—back-calculation of the original exposure pattern from measurements taken at different biological clocks requires temporal deconvolution as part of the PBPK framework.^43^ The UES is not a modelling exercise applied after data collection. It is the common dosimetric language without which multi-matrix and cross-cohort integration is arithmetically impossible—a hair measurement from Stockholm and a blood measurement from Nairobi cannot enter the same model without it.

Toxicokinetic modeling also changes what can be inferred from individual matrices in a longitudinal setting. For rapidly cleared compounds, PBPK-informed back-calculation links transient biomarkers to specific recent exposure events captured through concurrent environmental monitoring.^6^ For persistent compounds such as PFAS or organochlorines, repeated sampling over time is necessary to separate ongoing external exposure from legacy body burden that accumulated years earlier.^33^ External monitoring, multi-matrix biomonitoring, and toxicokinetic normalization are not independent research programs. They are components of a single study design, and the causal evidence that the exposome field needs is dependent on deploying them together.

## The Temporal Dimension: High-Throughput Infrastructure for Lifelong Trajectories

Chemical exposure is not a static property of an individual—it changes continuously, sometimes dramatically, over hours and days.^6,24^ Cross-sectional study designs cannot account for this variability, which is why the temporal dimension of the exposome is as important as its chemical breadth. Two things are needed simultaneously: biological matrices that retrospectively document past exposure, and frequent prospective sampling to capture ongoing dynamics. Segmental hair and nails serve the first function well, when available. Analyzed in segments corresponding to defined growth periods, they reconstruct monthly-resolution exposure histories months or years prior to sample collection^10,49^ a retrospective record that no prospective sampling scheme can replicate, because the body produced it continuously regardless of whether a study was running. However, the scope of captured exposures remains constrained by the physicochemical properties of the analytes and the specific mechanisms of their biological accumulation.

Prospective high-frequency sampling is harder to achieve in practice. Clinic-based biobanking carries significant participant burden and logistical costs^3^ that limit sampling to a handful of time points per year at best, which is far too coarse to capture episodic or rapidly fluctuating exposures. Microsampling technologies change this substantially. Beyond dried blood and urine spots, the microsampling landscape now includes volumetric absorptive microsampling,^50^ microneedle-based interstitial fluid collection, and wearable patch formats, each offering distinct coverage across matrices and analyte classes.^51^ Volumetric blood microsamplers and dried matrix spot devices^51^ applicable to blood, urine, and other liquid matrices—allow participants to self-collect samples at home with a finger prick or small swab. Dried blood spots (DBS) and dried urine spots (DUS)^52^ improve sample stability under ambient conditions for many compound classes, substantially reducing cold-chain storage requirements and, with them, the infrastructure costs that have historically limited longitudinal exposome research to well-resourced settings. Microneedle patches for continuous interstitial fluid monitoring^36,53^ go further still, offering temporal resolution that discrete sampling cannot approach (Figure 4).

**Figure 4:**
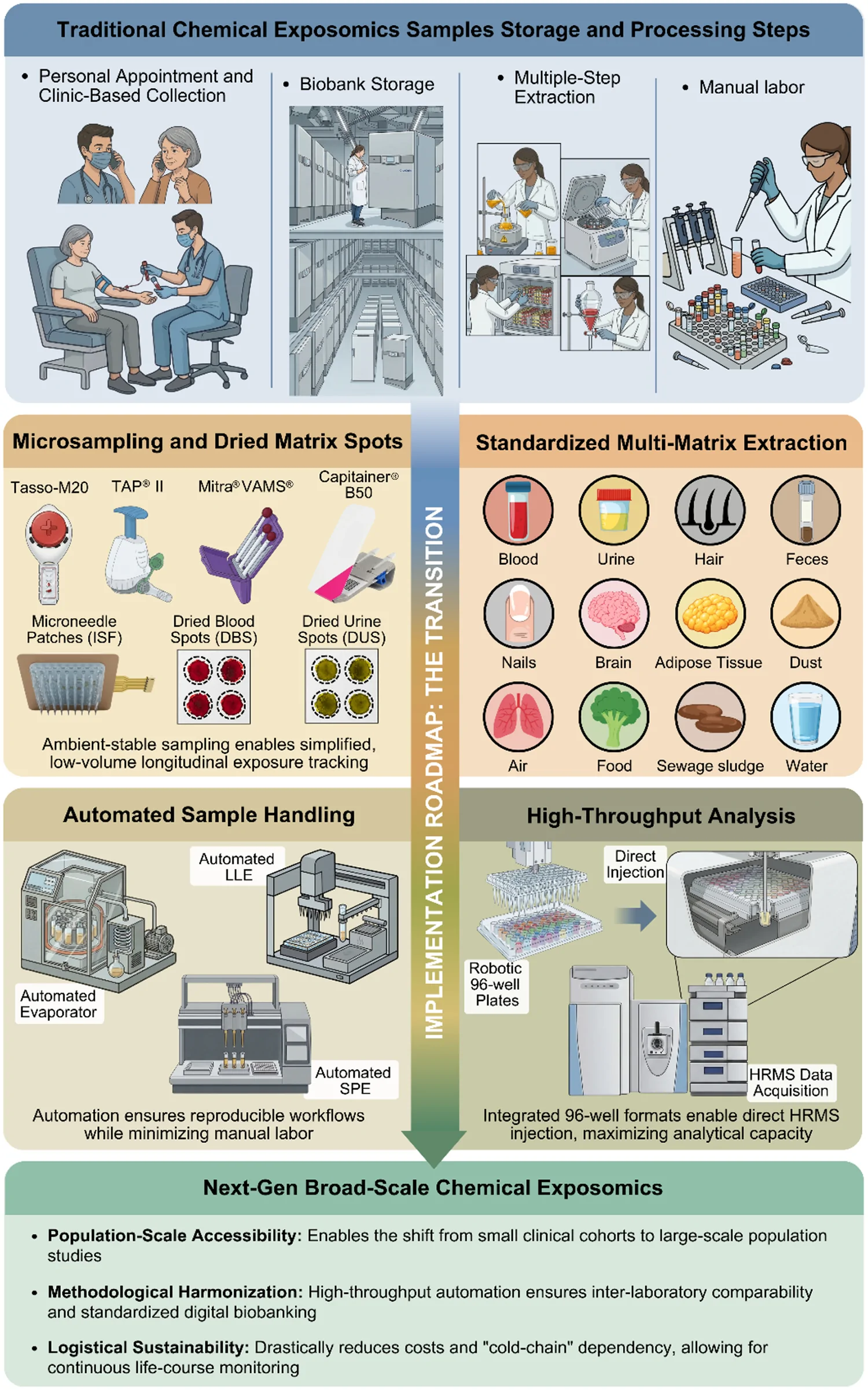
Implementation roadmap for high-throughput population-scale chemical exposomics. The transition from clinic-based collection and serial manual processing toward ambient-stable microsampling and automated multi-matrix workflows is shown. Microsampling technologies including dried blood spots (DBS), dried urine spots (DUS), and interstitial fluid microneedle patches eliminate cold-chain dependencies and enable home-based longitudinal sampling. Automated liquid-handling systems and 96-well plate formats ensure reproducible extraction conditions across samples, sites, and time points, supporting the methodological harmonization required for cross-cohort integration.

Collecting samples at scale is only half the problem—processing them is the other. Individual sample extraction by hand is too slow, too variable, and too labor-intensive to support the throughput needed (Figure 4).^54,55^ Transition to automated liquid-handling systems and 96-well plate formats is not just an efficiency issue—automation enforces identical extraction conditions across every sample, site, and timepoint, eliminating the operator-dependent variability^13^ that is invisible within a single study but becomes a serious confound when integrating data across cohorts and countries. Without this level of standardization, cross-cohort data pooling of the kind the GHEP envisions remains analytically out of reach.^32^

A further practical advantage of microsampling is the elimination of cold-chain infrastructure. Conventional biobanks are expensive to run, energy-intensive, and dependent on continuous refrigeration^51^ requirements that exclude most research settings outside well-resourced institutions. Stabilized dried samples stored at ambient temperature^52^ remove these dependencies entirely, which matters considerably if longitudinal exposome monitoring is ever to become viable at global scale.

From a toxicological perspective, high-frequency sampling is not simply better resolution—it may be the difference between detecting a causative exposure and missing it entirely. Many molecular initiating events are triggered not by chronic average concentrations but by acute concentration peaks of sufficient magnitude and duration to saturate detoxification capacity or overwhelm DNA repair.^56,57^ A blood sample collected days or weeks after such a spike records background levels and provides no evidence of the chemical that caused the event. Resolving this requires sampling dense enough to catch the spike while it is present.

## Navigating the Molecular Dark Matter: Mapping the Total Chemical Space

Knowing which matrices to sample and at what frequency addresses two of the three central challenges in chemical exposomics. The third—identifying what is actually present in those samples—is harder to solve. In typical LC-HRMS plasma datasets, annotation rates rarely exceed 5% at confidence level 2 or above.^4,9^ The remaining signals are detected but chemically uncharacterized, constituting what has been called the molecular dark matter of the exposome^58^ (Figure 5)—the large fraction of detected mass spectrometric features that remain chemically unidentified despite being consistently reproducible across samples. Getting beyond predefined target lists requires NTS workflows that profile the full chemical content of a sample rather than confirming the presence of a predetermined set of compounds. Full coverage across the polar-to-nonpolar spectrum requires complementary chromatographic platforms—reversed-phase LC (RPLC), hydrophilic interaction chromatography (HILIC), and gas chromatography (GC)^59,60^ since no single separation mode captures the entire relevant chemical space.

**Figure 5:**
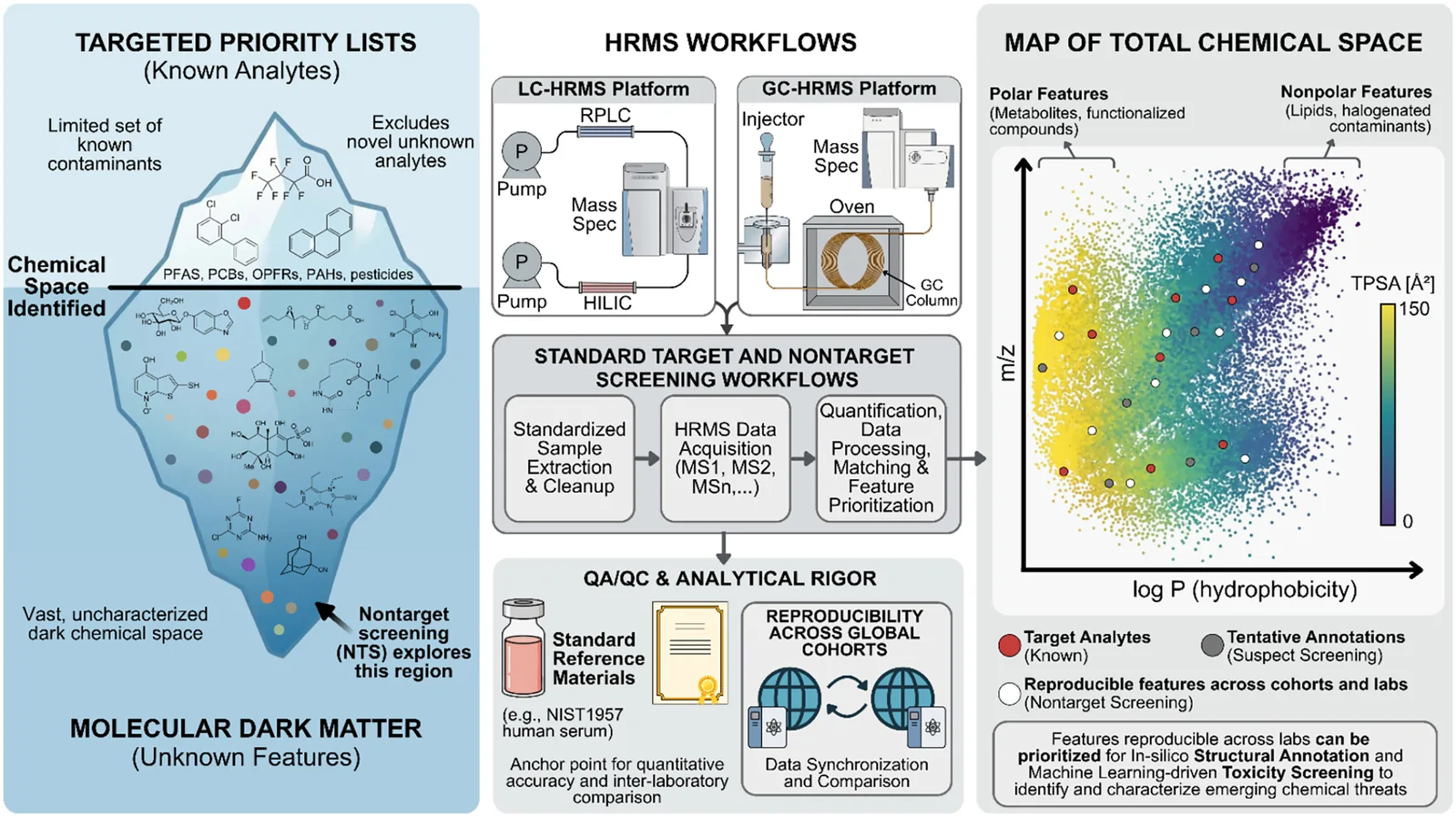
Analytical framework for mapping the molecular dark matter of the exposome. Target analysis covers only a small fraction of detected chemical features (iceberg tip), leaving a vast uncharacterized chemical space. Comprehensive characterization requires integrated HRMS workflows across complementary LC-HRMS (RPLC and HILIC) and GC-HRMS platforms, standardized for nontarget screening and benchmarked using standard reference materials such as NIST 1957 human serum. The multidimensional chemical space map coordinates features by mass-to-charge ratio (*m*/*z*) and hydrophobicity (log *P*), color-coded by topological polar surface area (TPSA). Reproducible features are prioritized for in silico structural annotation and machine-learning toxicity screening, with formal identification via reference standards triggered by hazard prioritization output.

Broader analytical coverage only translates into useful data if the underlying measurements are reproducible across laboratories and cohorts. An NTS feature detected in one instrument in Stockholm that cannot be confirmed on a different instrument in Boston—because extraction protocols differ, ionization conditions vary, or no shared reference material was used—is not evidence of chemical exposure. Standard Reference Materials such as NIST 1957 human serum^61^ provide the common analytical anchor that makes interlaboratory comparison meaningful. With harmonized methods and reference materials in place, a feature reproducibly appearing across cohorts in Stockholm, Boston, and Shanghai can be treated as a real signal rather than a platform-specific artifact (Figure 5). Beyond anchoring quantitative accuracy, standard reference materials serve a broader harmonization function: when analyzed consistently across laboratories and platforms, they allow systematic characterization of interlaboratory variability, propagation of measurement uncertainty, and identification of platform-specific biases that would otherwise confound cross-cohort comparisons. As Schymanski et al.^31^ argue, open collaborative data sharing and FAIR deposition of spectral libraries and annotated datasets are essential infrastructure for this—data quality frameworks that document sources of variability are as integral to the proposed design as the sampling protocols themselves.

When target, suspect, and nontarget measurements are plotted together in a common chemical space—coordinates such as *m*/*z* versus log *P*—the distribution of detected features across that space reveals patterns that no individual analytical strategy uncovers alone. Rather than attempting annotation of every detected feature, which spreads resources thinly across signals most of which will not reappear in independent datasets, features showing high reproducibility across cohorts and laboratories are selected for deeper characterization.^6,33^ This prioritization by reproducibility inverts the current default and focuses identification capacity where it is most likely to produce durable findings (Figure 5). Reproducibility thresholds can be defined empirically—requiring detection in more than 70% of samples within a cohort and confirmation in at least one independent dataset before a feature enters the prioritization queue. This is not a conservative compromise; it is a deliberate reallocation of finite identification capacity toward the signals most likely to persist across studies.

For these prioritized features, two tracks run in parallel. The first applies in silico structural annotation and machine-learning toxicity screening^46,62^ to estimate physicochemical properties—log *P*, p*K*_a_, intrinsic clearance—that feed into toxicokinetic models and allow internal dose reconstruction even before molecular identity is confirmed.^43^ This pipeline is iterative rather than simultaneous. NTS identifies reproducible features across cohorts; semi-quantitative approaches^63,64^ ionization efficiency prediction and internal standard-based relative quantification^54^ provide approximate concentration estimates sufficient for ranking and prioritization before identity is confirmed. Targeted quantification with reference standards follows for the highest-priority features, at which point UES translation becomes fully quantitative. For unconfirmed features earlier in the pipeline, semi-quantitative estimates carry higher uncertainty, but this is an accepted limitation of the discovery phase rather than a barrier to applying UES translation between samples—the goal at this stage is ranking exposure magnitudes and directing identification resources toward the most concerning signals, not replacing confirmed quantification.

This is not proposed as a route to regulatory decisions in isolation. Its role is to flag the highest-risk unknowns based on predicted toxicity and estimated internal dose, directing formal identification resources toward the signals most likely to matter.^65^ The second track—structural confirmation against reference standards—is triggered by that output. Together they form a pipeline capable of advancing from initial detection to regulatory-grade evidence^16,32^ for the most concerning emerging chemical threats, without requiring the field to formally identify every signal in the dark matter space (Figure 5).

NTS chemical data interpreted without biological context remains descriptive. Integrated with multi-omics profiles and contextual metadata including lifestyle and socioeconomic factors,^40,66,67^ it becomes mechanistically informative—linking specific chemical fingerprints to molecular perturbations^68^ before formal identity is even established. That is what converts a list of reproducible unknown signals into testable hypotheses about which chemicals are perturbing which biological pathways, and in whom (Figure 5).

## Toward a Global Human Exposome Project

Not all of the components described here are equally ready. Microsampling and automated extraction workflows are still being validated across matrices; chemical stability in dried microsamples remains compound- and time-dependent,^51^ and systematic studies on chemical stability across microsampling formats are a critical prerequisite for broad implementation. Self-collection strategies face non-technical barriers too—participant compliance, sampling discomfort, and user acceptability—and venipuncture remains the standard in many clinical and epidemiological settings.^69^ High-throughput PBPK normalization at the scale of thousands of NTS features is computationally tractable but has not yet been implemented routinely. Toxicokinetic parameters are unavailable for the majority of NTS-detected compounds, making UES translation approximate across much of the chemical space. Standardized multi-matrix NTS protocols remain limited, and interlaboratory comparability and annotation reproducibility are still being actively addressed by the community.

Implementing this framework at population scale will require coordinated investment in sampling infrastructure, analytical capacity, and data management systems. The transition to microsampling-based longitudinal collection reduces per-participant cost and logistical burden relative to conventional biobanking, though pilot studies demonstrating feasibility across the full matrix inventory will remain a necessary step before large-scale deployment. Such studies will be essential for testing the underlying hypotheses of the framework, identifying unforeseen practical constraints, and refining the proposed approach – particularly for components that remain underdeveloped in the current literature, such as high-throughput PBPK normalization across large NTS datasets and standardized multi-matrix collection protocols. Realizing the full potential of toxicokinetic integration across heterogeneous exposure data will additionally require close collaboration with experts in mathematics, bioinformatics, and computational modeling. International data sharing raises governance and privacy challenges that differ across jurisdictions. Coordinating these efforts across regions will require the institutional commitment that Vermeulen and Safarlou argue is currently absent from most large-scale exposome proposals.^30^ Studies built on this framework will also remain observational; richer longitudinal data with source attribution reduce confounding substantially, but do not eliminate it. None of this is a reason to delay defining the design framework—these are practical and technical problems with partial solutions already in development, not fundamental barriers to the approach itself.

The Global Human Exposome Project, established in 2025,^3,32^ is the institutional vehicle most capable of coordinating the infrastructure needed (Figure 2). Foundational initiatives such as iChemAtlas have begun characterizing the internal chemical landscape at unprecedented scale,^33^ but their necessarily constrained initial scope makes the case for multi-matrix longitudinal extension rather than weakening it. Three design choices would substantially improve the GHEP’s capacity to generate evidence that goes beyond chemical cataloging. First, cohorts could prioritize the multi-matrix sampling inventory described here, with particular attention to studies already archiving biological material—hair, teeth, placental tissue—that enables retrospective longitudinal reconstruction without additional participant burden. Second, the UES could be adopted as a common dosimetric language for cross-cohort data integration, replacing the matrix-specific concentration reporting that makes pooled analysis arithmetically incoherent. Third, NTS data from all matrices could be deposited in open repositories with harmonized metadata,^70^ allowing retrospective integration across studies not originally designed to be compatible.

There is also an equity dimension worth stating explicitly. The infrastructure constraints that have historically confined longitudinal exposome research to well-funded institutions—clinical sampling, cold-chain biobanking, centralized processing—are precisely the ones this framework is designed to remove. Ambient-stable microsampling eliminates refrigeration dependencies; automated extraction reduces per-sample cost; open harmonized repositories lower the barrier to data contribution and reuse. A GHEP built around these components could generate exposure data from populations in low- and middle-income settings that current biomonitoring infrastructure systematically excludes—the same populations that often bear the highest chemical exposure burdens.^1^ Concurrently, many molecular initiating events are driven not by chronic average concentrations but by short-duration peaks—organophosphate pesticides during application seasons, PAH spikes from episodic combustion events, phthalate bursts from acute product use. High-frequency sampling is what makes these toxicologically relevant exposure patterns observable rather than averaged away.^56,57^

The case for defining this architecture now rather than later is straightforward. As Martin notes in discussing the field’s readiness for a large-scale exposome initiative,^26^ the analogy to the Human Genome Project is instructive—that project launched before sequencing technology was mature, and methods improved throughout. Waiting for every component to be fully optimized before committing to a design framework means the infrastructure being built now will embed the same limitations that have constrained the field for two decades. High-frequency multi-matrix monitoring does not improve instrument sensitivity—it changes what the study design can observe: the episodic spike that cleared before the clinic visit,^6,25^ the source attribution that a single matrix cannot support,^24^ the molecular initiating event that preceded symptoms by weeks.^56^ Synchronizing chemical and multi-omics measurements in time^71^ is what transforms a catalog of detected exposures into mechanistic evidence about which compounds drive which biological changes. That capacity does not yet exist—not because the tools are insufficient, but because no study has deployed them together in a single integrated longitudinal design.^6,24,25^ The three design principles described in this perspective are not independently sufficient: without multi-matrix sampling, toxicokinetic normalization has too little to work with without concurrent external monitoring; internal dose estimates have no source to point to. As Miller et al.^27^ argue, measurements become substantially more powerful when integrated with process-based models of internal dose—which is precisely what the UES provides. Emerging international infrastructure efforts—including EIRENE^72^ in Europe, NEXUS^73^ in the United States, and coordinating bodies such as IHEN^74^ and the Global Exposome Forum^75^ are actively building the institutional foundations that any large-scale exposome initiative will require. The design principles proposed here are meant to complement those efforts by specifying what the sampling, normalization, and prioritization layer of any large-scale exposome initiative should look like. Reconstructing individual chemical trajectories from environmental source through biological dose to molecular perturbation^7^ is the design condition the field has been working toward since the exposome was first proposed. The GHEP is the opportunity to finally build it.

## Data Availability

This Perspective contains no primary data. All data discussed are available through the original publications cited.
